# Transcutaneous Electrical Nerve Stimulation and Aerobic Exercise for Alcohol-Related Peripheral Neuropathy: A Case Report

**DOI:** 10.7759/cureus.76338

**Published:** 2024-12-24

**Authors:** Yuto Niwa, Manabu Nankaku, Ryosuke Ikeguchi

**Affiliations:** 1 Rehabilitation Unit, Kyoto University Hospital, Kytoto, JPN; 2 Rehabilitation Unit, Kyoto University Hospital, Kyoto, JPN; 3 Department of Rehabilitation Medicine, Kyoto University Graduate School of Medicine, Kyoto, JPN

**Keywords:** aerobic exercise, alcohol-related peripheral neuropathy, neuropathic pain, numbness, transcutaneous electrical nerve stimulation

## Abstract

Alcohol use disorders can cause peripheral and central neurological disorders with symptoms such as pain, numbness, paresthesia, and dysesthesia, often impairing walking ability. However, effective treatments for alcohol-related peripheral neuropathy are yet to be identified. This case report highlights the successful use of dysesthesia-matched transcutaneous electrical nerve stimulation (DM-TENS) and aerobic exercise in a 53-year-old woman with alcohol-related peripheral neuropathy who presented with severe pain and walking difficulties. Initially, her pain and numbness were scored 8-9 on the numerical rating scale (NRS), and her timed up-and-go test (TUG) time was 23.00 seconds. DM-TENS reduced her symptoms (NRS: 3), and the introduction of aerobic exercise further enhanced her recovery. After six days, her pain and numbness improved (NRS: 5), and her walking ability markedly increased (TUG: 10.94 s). By day 10, her symptoms had significantly resolved (NRS: 1). This case suggests that combining DM-TENS and aerobic exercise can effectively reduce pain, improve mobility, and offer a promising therapeutic approach to alcohol-related peripheral neuropathy.

## Introduction

Alcohol use disorders are known to cause a variety of peripheral and central neurological disorders, including peripheral neuropathy, ataxia, confusion, and cognitive impairment. Among these conditions, 44% of individuals with chronic alcohol abuse exhibit signs and symptoms of peripheral neuropathy. Alcohol-related peripheral neuropathy is characterized by pain, numbness, paresthesia, and dysesthesia [1]. These symptoms typically begin distally. In addition, pain and numbness caused by peripheral neuropathy tend to occur in the lower limbs and can limit the patient's day-to-day walking ability [2]. Therefore, rehabilitation strategies specifically designed to improve pain and numbness are required for these patients.

Pain associated with peripheral neuropathy is classified as neuropathic pain [3]. While pharmacotherapy is an effective therapeutic approach for pain, numbness, and dysesthesia, the degree of improvement is generally modest and is often accompanied by a high risk of adverse events [4]. Spinal cord stimulation (SCS) is a non-pharmacological intervention for pain and numbness. However, as SCS involves surgery, patients cannot easily choose this treatment. In contrast, aerobic exercise and transcutaneous electrical nerve stimulation (TENS) are considered noninvasive treatments for neuropathic pain, given their ability to activate the descending pain inhibitory system [5]. These treatments are not associated with a high risk of serious adverse events and can be easily implemented. However, no published studies have evaluated the efficacy of aerobic exercise combined with TENS in treating peripheral neuropathy.

In this report, we describe a case in which a combination of TENS and aerobic exercise was used to treat pain and numbness associated with alcohol-related peripheral neuropathy.

## Case presentation

Patient information

A 53-year-old Asian female patient presented with lower limb pain and numbness for the past two years. Despite a history of the use of Ca2+ channel a2d ligands, anticonvulsants, and physical therapy, the chief complaints were severe pain, numbness in the lower limbs, and gait disturbances. Neurological examination revealed decreased vibration sensation and light touch perception in the lower extremities, particularly distal to the ankles. Pinprick sensation was reduced below the ankles, indicating sensory deficits consistent with peripheral neuropathy. Proprioceptive deficits were suggested by a positive Romberg test. Fursultiamine hydrochloride and methyl cobalt were administered three times per day.

Clinical findings

Subjective pain intensity and numbness were measured before treatment and at discharge (five days after treatment). A numerical rating scale (NRS) of 0-10 was used to describe the pain intensity and numbness; scores ranged from 0 (no pain/no numbness) to 10 (maximal pain/maximal numbness). The Douleur Neuropathique 4 Questionnaire (DN4) was used to screen for neuropathic pain [6]. The Central Sensitization Inventory-9 (CSI-9) assessed central sensitization-related symptoms. The CSI-9 consists of nine items and is a short version of the 25-item CSI [7]. A total score of 0-9 is classified as “subclinical,” 10-19 is classified as “mild,” and 20-36 is classified as “moderate/severe.” Kinesiophobia, or the fear of movement, was assessed using the Japanese version of the previously validated Tampa Scale for Kinesiophobia-11 (TSK-11) [8]. TSK-11 scores ≥ 25 were considered indicative of excessive kinesiophobia [9]. The TSK-11 consists of 11 items and is a short version of the 17-item TSK. The Pain Catastrophizing Scale-6 (PCS-6) was used to assess pain catastrophizing. The PCS-6 consists of six items and is a short version of the 14-item PCS [10]. The Pain Self-efficacy Questionnaire-4 (PSEQ-4) was used to assess self-efficacy for pain. The PSEQ-4 consists of four items and is a short version of the 10-item PSEQ [11].

Physical performance was assessed using the five-repetition chair standing test (CST-5), 10-minute walk test (10 MWT), and timed up-and-go test (TUG), as has been done in previous studies [12]. The low-threshold mechanoreceptive function was assessed using Von Frey filaments (Semmes-Weinstein; Sakai Iryou Co., Tokyo, Japan).

The local tactile threshold was measured using the method of limits applied to the foot. Monofilaments were applied in the order of decreasing force until the patient continued to report tactile sensation. Measurements were obtained at the lowest filament for which tactile sensation was felt. Tactile and painful sensations were assessed using tissue paper and needles. An NRS of 0 to 10 was used to score the intensity of tactile and pain sensation, with an NRS score of 0 indicating “no touch/no pain” and 10 indicating “as much as touch to the forearm/as much as pain to the forearm”). These assessments were performed at admission and discharge (Table 1).

**Table 1:** Timeline of the medical care administered to the patient NRS, numerical rating scale; DN4, Douleur Neuropathique-4 questionnaire; CSI-9, Central Sensitization Inventory-9; TSK-11, Tampa Scale for Kinesiophobia-11; PCS-6, Pain Catastrophizing Scale-6; PSEQ-4, Pain Self-efficacy Questionnaire-4; CST-5, five-repetition chair standing test; 10 MWT, 10-minute walk test; TUG, timed up-and-go test.

	Baseline		Follow-up (Intervention＋6 day)	Follow-up (Intervention＋10 day)
	Before TENS	After TENS		
Pain (NRS, 0–10)	8	3	5	1
Numbness (NRS, 0–10)	9	3	5	1
DN-4 (score)	7		4	
CSI-9 (score)	25		23	
TSK-11 (score)	32		25	
PCS-6 (score)	11		13	
PSEQ-4 (score)	5		8	
CST-5 (seconds)	none		27.06	
10 MWT (seconds, step)	19.63, 29		10.47, 19	
TUG (seconds)	23.00		10.94	
Tactile threshold (right/left, g)	2.0/2.0		0.4/0.4	
Tactile sensation in the dorsal foot (NRS, 0–10; right/left)	1/1		5/5	
Tactile sensation in the plantar region (NRS, 0–10; right/left)	1/1		5/6	
Painful sensation in the dorsal foot (NRS, 0–10; right/left)	1/0.5		2/2	
Painful sensation in the plantar region (NRS, 0–10; right/left)	3/0.5		3/6	

Therapeutic interventions

TENS (Espurge; Ito Physiotherapy & Rehabilitation Co., Ltd., Tokyo, Japan) was performed with a continuous pulse pattern and a pulse duration of 50 μs. The stimulation intensity and frequency were matched to the numbness perception of the patient using dysesthesia-matched TENS (DM-TENS), as described by Nishi et al. [13]. We attached 5-cm^2^ self-adhesive electrodes (Axelgaard; Ito Physiotherapy & Rehabilitation Co., Ltd.) at the dorsum and the sole of the foot.

A stationary cycle ergometer (Aerobike 75XL III; Konami Sports Life, Kanagawa, Japan) was used for aerobic exercise. The patient cycled for 10-15 minutes at the preferred intensity ranging from 30-50% heart rate reserve (HRR) [14]. The saddle and handlebars were positioned according to the preferences of the patient, and they were instructed to place their hands on the handlebars or grip them during exercise.

Follow‐up and outcomes

The patient progression is shown in Table 1. The patient experienced severe pain, numbness, dysesthesia with pain catastrophizing, fear of movement, and decreased self-efficacy. In addition, physical function was severely impaired, with a 10 MWT time of 19.63 seconds, a TUG time of 23.00 seconds, and an “unable to stand”-status on the CST-5. The patient reported decreased pain and numbness immediately after TENS. After five days of TENS and aerobic exercise, the intensity of pain and numbness decreased from severe (NRS score: 8-9) to moderate (NRS score: 5). Furthermore, the pain catastrophizing of the patient, fear of movement, low self-efficacy, and impaired physical function (10 MWT time: 10.47 seconds, TUG time: 10.94 seconds, CST: 5:27.06 seconds) improved. After 10 days of follow-up, the intensity of pain and numbness decreased from moderate (NRS score: 5) to mild (NRS score: 3).

Perspective of the patient

Before TENS, the patient reported: “I am afraid to walk because I have no sensation under my ankle, and I do not know what it feels like to walk. I have trouble with numbness and pain.” During TENS, the patient’s report changed to: “I do not remember what it feels like to have numb feet. They feel like normal feet now.” After TENS, the patient reported: “My feet feel better with respect to the numbness, and it is easier to walk.” On the sixth day of the intervention, the patient said: “I can feel my feet better than before. My numbness and pain are better, and my fear of walking has decreased.” After discharge from the hospital, she called the hospital and said, “I can do household chores without difficulty, and my father told me 'You can walk normally now.' I was happy to hear this. I will continue to exercise.”

## Discussion

In this case report, we investigated the effects of DM-TENS and aerobic exercise on pain and numbness in a patient with alcohol-related peripheral neuropathy. The results showed immediate hypoalgesia and an improvement in numbness with TENS alone. When TENS was combined with aerobic exercise and continued, hypoalgesia and improvement in numbness were observed after the intervention. Additionally, the ability to walk had greatly increased. TENS has been widely used for various neuropathic conditions with variable success. This case report demonstrates the potential benefit of combining DM-TENS with aerobic exercise to improve walking ability and reduce pain and numbness in a patient with alcohol-related peripheral neuropathy.

In this case, the patient experienced numbness and pain due to alcohol-induced peripheral neuropathy, and significant improvement was observed with DM-TENS. Nishi et al. described the selective blockade of sensory nerves specific to numbness by tuning electrical stimulation to numbness [13]. Furthermore, TENS reduces pain by activating large peripheral afferent fibers that send inputs to the central nervous system and activate descending pain-inhibitory systems associated with neural activity in the periaqueductal gray, rostral ventromedial medulla, and spinal cord [15]. In addition, the gate control theory proposed by Melzack and Wall in 1965 suggests that non-nociceptive stimulation of low-threshold, non-nociceptive, large-diameter Ab fibers activates inhibitory interneurons, thereby suppressing the conduction and discharge of nociceptive Ad and C fibers in the dorsal horn and transmitting to the central cortex [16]. These hypoalgesic mechanisms likely improved the pain and numbness of the patient when using DM-TENS.

In this case, cycling exercises were performed after numbness entrainment using TENS. Cycling was less likely to induce fear of movement or is associated with a lower risk of falling compared to walking in patients with sensory impairment; this patient could safely complete the exercise. In this case, the aerobic exercise was set at an intensity equivalent to the low-intensity exercise recommended by the American College of Sports Medicine [17]. Low-intensity exercise can produce hypoalgesic effects [14]. The activation of the endogenous opioid and serotonin systems causes exercise-induced hypoalgesia [18]. Furthermore, myokine release associated with muscle contraction has an anti-inflammatory effect on neuroinflammatory diseases such as alcohol-induced peripheral neuropathy [18,19]. Thus, the pain and numbness in this case may have improved due to exercise-induced secretion of endogenous opioids and myokines.

The patient had difficulty walking because of kinesiophobia caused by pain and numbness. The patient developed thoughts of fear avoidance due to sensory abnormalities, which led to inactivity and impaired physical function. We believe that this patient overcame this fear of movement by improving pain, numbness, and hyposensitivity with DM-TENS and cycling exercises. In this case, the TSK-11 showed an improvement of seven points, which is much greater than in a previous study [20], in which patients with chronic pain showed a change of 5-6. Improvement in fear of falling may have further facilitated exercise therapy by breaking the negative cycle of fear-avoidance thoughts, significantly improving walking ability.

This was only a single case study, which limits the generalizability of our results. In addition, there were no follow-up data; therefore, it is unclear whether these effects would persist over time. Moreover, as this case study did not include sham stimulation, a placebo effect cannot be excluded. Despite these limitations, this case study provides valuable insights into the effective treatment of alcohol-related peripheral neuropathy. Further validation through placebo-controlled prospective studies is necessary.

## Conclusions

Peripheral neuropathy is a common complication of alcohol-related disorders, often causing severe pain, numbness, and impaired mobility. This case report highlights the potential efficacy of combining DM-TENS and aerobic exercise as non-pharmacological treatments. DM-TENS was effective in alleviating sensory disturbances by modulating peripheral nerve activity, while aerobic exercise promoted hypoalgesia and improved physical function. Together, these interventions substantially reduced the patient’s symptoms and enhanced walking ability. Further studies are needed to confirm the broader applicability and long-term effects of this approach.
